# Green Landscapes of Care: The Potential of Gardens to Support the Well-Being of Asylum Seekers in Ireland

**DOI:** 10.3390/ijerph22091410

**Published:** 2025-09-10

**Authors:** Felicity Daly, Sally Ann Lenehan, Jacqui O’Riordan

**Affiliations:** 1 Trinity Centre for Global Health, Trinity College Dubin, Dublin 2, Ireland; 2 School of Public Health, University College Cork, T12 XF62 Cork, Ireland; 3 Independent Researcher, Leap, Co Cork, Ireland; 4 School of Applied Social Studies, University College Cork, T12 HY8E Cork, Ireland; jacquior@ucc.ie

**Keywords:** migration, feminist ethics of care, mental stress, well-being, green space, horticultural therapy

## Abstract

Engaging vulnerable migrants in nature-based activities demonstrates that access to green space can provide a safe place to process trauma, allowing vulnerable forced migrants to enhance their sense of subjective well-being, to breathe and to be. Framed by the feminist ethics of care concept of ‘universal care’, this qualitative study utilised semi-structured interviews, focus group discussion and participant observation to explore asylum seekers’ opportunities for giving and receiving care for people and planet in green spaces outside of institutional international protection accommodation, particularly among those who have access to community gardens. This research contributes to understanding the multigenerational benefits of green space and the potential of forms of horticultural therapy to support the health and well-being of vulnerable forced migrants of all ages. This research has implications for how care for international protection applicants could be enhanced in Ireland and elsewhere through expanding access to safe and inclusive green spaces. It provides a model of a landscape of care support mitigation of pre- and post-migration trauma and mental stress.

## 1. Introduction

Globally, one in eight people migrate due to factors including economic, political, demographic and environmental challenges, with increasing levels of conflict, violence, persecution and human rights violations driving forced migration [1]. Forced migrants requiring international protection, such as refugees and asylum seekers, continue to face unique challenges after arrival in a reception country. There is a growing commitment within reception countries to improve the health of forced migrants and address the root causes that negatively influence their health [2].

The Irish system to accommodate asylum seekers who have applied for international protection, officially titled Direct Provision and Dispersal (DP), allocates places in centres located around the county and can relocate people to other centres on short notice. These congregated institutional settings provide a single room per family and shared rooms for individuals and often limited access to cooking facilities where meals are catered. Critiques of the spaces inside of DP are concerned about a range of care deficits, overcrowding, poor hygiene [3] and lack of privacy in an institutional hierarchy wherein residents are constantly surveilled [4]. Many DP residents experience poor mental health outcomes, such as elevated levels of self-reported post-traumatic stress disorder, depression, anxiety and the precursors of suicidality associated with the length of time spent waiting for a decision on an international protection application [5]. The health of children living in DP may be compromised by less than optimum utilisation of general practitioner services compared to children born to EU citizens [6]. Child development is thwarted by limits of indoor and outdoor space or separate spaces for children growing up in DP which obstructs opportunities for play [7,8].

Spatial limitations impact negatively on the health and well-being of forced migrants in a variety of settings. The shrinking physical spaces available to Rohingya refugees in Southeast Asia erode ‘the psychological margins of trust and hope in their new host country’ [9]. Afghan migrants fleeing conflict often find shelter with multiple families sharing dwellings in Peshawar, Pakistan, wherein poor sanitation compromises hygiene and contributes to poor mental health [10]. Efforts to support children of forced migrants growing up in small, congregated settings have led to proposals such as repurposing maritime shipping containers to serve as classrooms to maintain educational continuity [11]. The problem of providing adequate accommodation for forced migrants persists in high-income countries, such as Canada, where Syrian families with several children share limited rooms [12] and immigration detention centres restrict undocumented migrants to small spaces and undermine their mental health through surveillance [13].

In 2021, the Irish government made a commitment to replace the DP system and adopt a human rights-based approach to international protection applicants, end the institutional model, provide ‘own door’ accommodation, improve community integration and deliver an enhanced model of community healthcare [14]. That vision did not explicitly consider asylum seekers’ access to green spaces. In a mostly rural country, asylum seekers in Ireland are often located in urban settings and may face barriers to accessing space outdoors as among migrants in other high-income countries [15]. Currently, there has been no progress on deinstitutionalisation and little evidence of implementation of efforts to improve access to health care and community integration.

There is increased focus on the role of nature-based interventions to support the health and well-being of forced migrants in reception countries [16]. Care ethicists have offered a vision of ‘universal care’ which implies that ‘we are all jointly responsible for hands-on care work, as well as engaging with and caring about the flourishing of other people and the planet’ [17,18]. The concept of ‘universal care’ provided a conceptual basis for this research which sought to explore asylum seekers’ opportunities for giving and receiving care for people and planet in spaces outside of Ireland’s international protection system, particularly among those who have access to a community garden.

This paper explores how care connects with asylum seekers’ access to green space and presents lived experiences of asylum seekers. The material presented in this paper is drawn from a three-year qualitative, participatory research project which sought to re-envision care relations in Ireland following the COVID-19 pandemic. The overall aim of the CareVisions project was to explore moral and ethical questions about the future of care, to inform future care policy debates about how we might envision ‘better care’ in Ireland and beyond. Empirical work within the project focused on the care networks and care experiences of two vulnerable groups in Irish society, disabled people and asylum seekers. The conceptual framing of this project is based in the feminist ethics of care scholarship [19] which recognizes the centrality of care to human and non-human life.

Prior to specific details on the reported study, we provide a literature review on green spaces and well-being including scholarly work with vulnerable populations.

### 1.1. Green Space and Well-Being

Positive associations between access to nature and health outcomes are well established, demonstrating that contact with nature increases individuals’ subjective well-being and reduces risk of conditions linked to chronic stress [20]. Access to nature and outdoor recreation can reduce the impact of exposure to environmental factors and promote restoration [20]. Recent systematic reviews confirm the positive benefits of contact with nature and nature-based interventions on mental health and mood outcomes [21] and boosting the immune response [22]. Healthy child development, psychological well-being and positive environmental attitudes are also enhanced when children spend time in natural environments [23].

Access to public green spaces, e.g., open, undeveloped land, urban parks and street greenery, is important given its beneficial associations with health benefits, such as all-cause and stroke-specific mortality, CVD morbidity, cardiometabolic factors, mental health, low birth weight, physical activity and sleep quality [24]. Evidence also suggests a positive relationship between access to green space within the built environment and self-reported well-being and stress reduction [25].

A recent systematic review confirmed that community gardens and gardening positively impact health outcomes amongst diverse populations and demonstrated positive associations between community gardening and mental health and well-being [26].

### 1.2. Benefits of Green Space for Vulnerable Migrants

A thematic review identifies that community gardens have the potential to enhance well-being amongst vulnerable populations both at the individual level and the relational and social level [27]. Vulnerable migrants, including refugees, participating in community gardening have experienced an increase in independence self-worth, a sense of belonging, and enhanced relationships and social connections [28,29]. Psychosocial benefits conferred to refugees and migrants through community gardens suggest that these landscapes create ‘a space for recovery and respite from stress’ where bonds with other vulnerable migrants can be nurtured, where there are opportunities to socialise with people from the wider community and where children have a safe space to play [16]. An earlier study suggests that refugee- and asylum-seeker-led food projects in Ireland, encompassing community gardens and other initiatives, “point to the possibility of new ways of political participation, self-organisation and of labour inclusion” [30].

Opportunities for the inclusion of all members of a community, regardless of the status conferred by a State, is important when considering who can access green spaces and how. All United Nations Member States are mandated to provide ‘universal access to safe and inclusive green and public spaces’ [31] by 2030 to achieve target 11.7 of the Sustainable Development Goals. Given that refugees and asylum seekers may have no autonomous choice about where and for how long they will reside in international protection accommodation, interventions may be required that can support their ability to benefit from contact with nature.

## 2. Materials and Methods

The CareVisions project’s empirical research used a constructivist epistemological perspective to understand care networks and experiences in order to promote ‘better care’ [19]. While we referred to the COREQ checklist in order to report our methodology, we have chosen to reflect our approach authentically [32]. This paper presents data collected from research with 10 asylum seekers resulting from purposive sampling among those currently or recently living in three DP centres located in different parts of Ireland. The data is drawn from semi-structured interviews (*n* = 4) and a focus group discussion (*n* = 6). Common demographics include adults age 18 and over, living in DP for over one year and raising at least one child. Table 1 provides further information on participants and research methods.

The key research question guiding our empirical work is how do asylum seekers give meaning to care in their lives both as receivers and givers of care. Topic guides for semi-structured interviews explored care experiences and networks. A focus group discussion guided asylum seekers to identify how care connects to what is going on in the community garden they have access to. The first author conducted 3 interviews, the third author conducted 1 interview and they co-facilitated the focus group discussion.

This paper incorporates perspectives from the first author’s participant observation conducted during Spring–Summer in 2022 and 2023. In addition to two research team members, the second author is a horticulturalist who was employed (2021–2023) by the Clonakilty Friends of Asylum Seekers as a support worker. Her perspectives provide insights into forms of care delivered with adults and children resident in the DP centre, their engagement in the community garden and the benefits they experience from horticultural therapy.

To pursue research within the Clonakilty Community Garden, the two research team members contacted the Clonakilty Friends of Asylum Seekers to ascertain interest in approaching DP residents about involvement with this project. They agreed to support respondent recruitment, host a space for focus group discussions and allow one of the researchers to engage in participant observation. A preliminary exploratory meeting held in May 2022 allowed for discussion of this study with potential research participants many of whom expressed interest in taking part in the research. All but one of these participants subsequently participated in the focus group discussion but other participants were secured for an interview.

Ethical approval for this research was granted through the University College Cork Social Research Ethics Committee. Ethical approval was only for participants over the age of 18 and thus children’s perspectives presented in this paper are conveyed as what was shared by parents and a support worker. Although we did not conduct research directly with children, we represent their experiences through the observations of our research participants. All fieldwork took place between March 2022 and June 2023.

Interviews and the focus group were recorded, transcribed and anonymised. The first author coded this data and their field notes from participant observation using an inductive approach to identify themes. Themes emerging from data analysis were discussed among the co-authors to determine the content that forms these findings. Table 2 details the research questions pursued in the various methods of data collection which generated the emergent themes.

### Study Context

The majority of the fieldwork (focus group, in-person interviews and participant observation) undertaken for this study was conducted within the Clonakilty Community Garden and this section describes the context of that setting. The strategy of dispersing international protection applicants around Ireland led to the opening in 2001 of a DP centre in a former holiday lodge in Clonakilty, a town of less than 5000 residents in rural County Cork [4,33]. Soon after, a group of volunteers established the Clonakilty Friends of Asylum Seekers, hereafter the Friends, to offer friendship and support to residents of the DP centre. The Friends are a registered charity, partly supported by Irish statutory funding for health promotion and prevention, employing part-time staff and engaging volunteers from the local community. Their activities provide practical and moral support to international protection applicants and there is a significant focus on care and support of children residing in the DP centre.

Many of the Friends’ activities are held within a community garden they established in 2014 on a piece of land opposite the DP centre. Initially set up with raised bed allotments providing a shared community space to grow seasonal produce, the garden facilitated a type of ‘food activism’ for residents of the DP centre and members of the local community [28]. At that time, gardening conferred a sense of purpose for asylum seekers while awaiting decisions on their applications for international protection. Following a Supreme Court challenge by an asylum seeker in 2017, Ireland now permits applicants for international protection to work following a 6-month initial wait [34,35].

**Figure 1:**
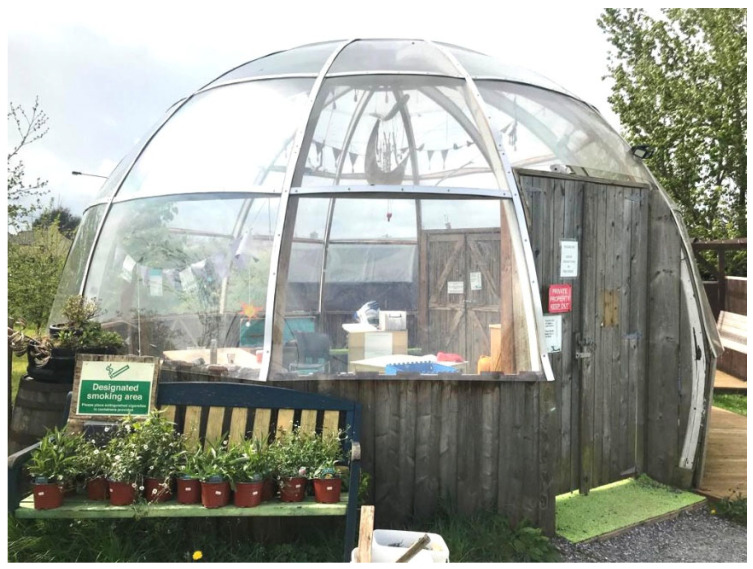
The garden dome at the Clonakilty Community Garden. Photo credit: Jacqui O’Riordan.

As custodians of the garden, the Friends extended it onto an adjacent piece of land, creating a multi-functional space that includes a large garden dome which offers year-round use of an indoor space, wholly independent of the DP centre—see Figure 1. Rain or shine, residents can take part in a range of care and support activities offered by the Friends’ support workers. These include a mother and baby group, well-being classes, workshops and one-on-one meetings with support workers. Such activities create opportunities for open discussion with residents about concerns they may have so that they can be signposted to available services. Group activities can help connect DP residents with each other and foster peer support, which single parents find particularly beneficial. An annual summer garden party is hosted by the Friends, inviting volunteers, community members and local stakeholders to join DP residents to enjoy music and taste world cuisine, fostering community integration.

**Figure 2:**
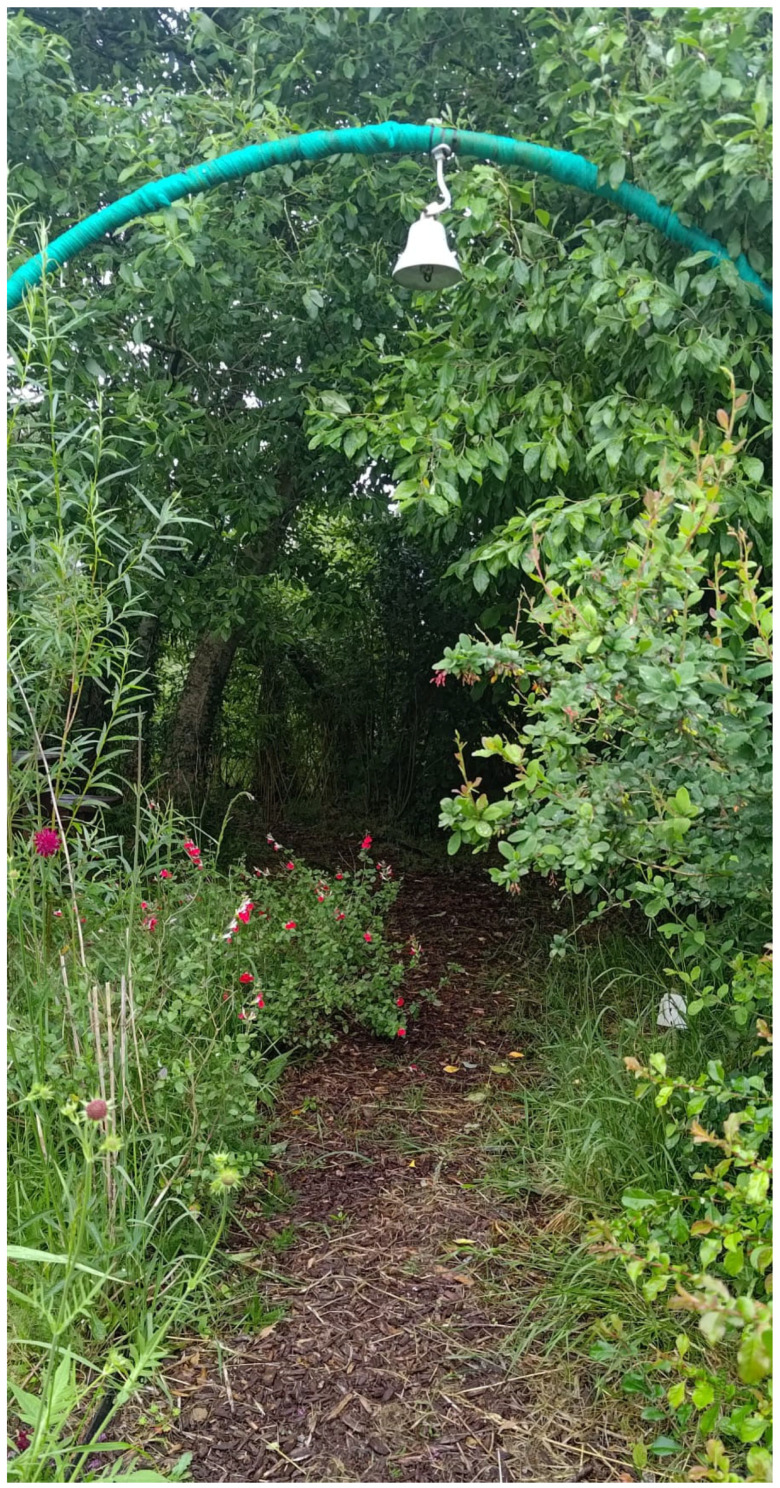
The sensory pathway in the Clonakilty Community Garden. Photo credit: Sally Ann Lenehan.

The garden is a space for children to play with others and to spend time, one-on-one, with Friends’ support workers responding to their care needs. Throughout the summer, the Friends offer a gardening club for children which draws on therapeutic concepts. Children can engage in activities like making bug hotels, learn about the importance of biodiversity and be introduced to a wider environmental protection perspective. In the spring of 2023, part of the Clonakilty Community Garden was set aside to establish a pathway along a section of the garden’s perimeter. This ‘sensory garden’ was designed with input from a Friends’ support worker who is an experienced horticulturalist incorporating principles of horticultural therapy into her work. It was planted with herbs, grasses, flowering plants and trees specifically chosen for their qualities that can awaken the senses of sight, smell, sound and touch—see Figure 2.

A group of children resident in the DP centre were involved in selecting, planting and maintaining the new pathway. The pathway leads to a traditional willow arch that creates a secluded corner that can enhance residents’ safe space for reflection and private conversations. The aim of the sensory garden was to maximise social, cognitive, physical and psychological functioning and/or to enhance general health and wellness for residents, particularly the children who regularly use the garden. The initiative was identified as a form of “garden therapy for those who have experienced trauma and for children with special needs” [36].

## 3. Results

### 3.1. Barriers and Facilitators for Green Space Access When Resident in DP

Explorations of asylum seekers’ experiences of care often centred on how space inside DP constructs care deficits but they also revealed how lack of access to space outside DP impacts health and well-being. Participant A, a man raising a son in DP, remarked on strategies to get out of the confines of their shared room. Referring to his accommodation, he said “It was a very small room. Just like a 10 by 10 room…it’s like a hard road around our building. So, the kids had no place to play…the only place that is near us it’s about 3 to 4 km away where we had to trek, because taking taxis will be quite expensive for us…the next time I said ‘oh, let’s go to that place’ [my son] said ‘maybe it will start raining or it’s too long, it’s too far’. We will still try many times to just [get out] with other residents”. When trips to that park were not feasible, he shared that his son “just goes outside the building, he will play around then I bring him back.”

Given that our research took place following the COVID-19 pandemic, challenges to health protection within the congregated settings of DP featured in some accounts. Following an outbreak of COVID-19 in his DP centre, Participant A was concerned about infection risks in the common areas. He instructed his son “stay here. Unless we are going out to do things, you’re not playing in this environment”. He and his young son self-isolated inside their small room for 60 days during a lockdown, without access to fresh air and outdoor recreation.

Some asylum seekers have had opportunities to engage in collective activities to care for the local environment through the Tidy Towns initiative, a voluntary programme which encourages community members to keep their streets free from litter and has a broader impact on local regeneration and gardening [37]. Participant B, a woman raising two sons, found participation in this activity enhanced her sense of integration into the local community nearby the DP centre where they lived at the time. She recalled “every Saturday, if anyone is free, we just go and do everything [collect litter, plant flowers]. And you feel proud yourself that I’m part of the community. And after we finish…we go and have a cup of tea. We have chats with people…sometimes [the local Tidy Towns committee] arrange dinner parties and we are also invited…We have also been invited to attend meetings because we are part of them. So, you feel that you’re also part of the community”. Eventually, Participant B and her sons were relocated to another DP centre far away and in their new location she missed that opportunity for connection outdoors which had provided opportunities to engage in a form of collective care for the environment and to make new connections with people.

A focus group discussion conducted with six women inside the Clonakilty Community Garden dome revealed other barriers to actively engaging in nature-based activities. Participant E, who was granted international protection and moved from the Clonakilty DP centre to the local community, remarked on how engagement of asylum seekers in gardening developed over time. She said “before when people were not working, we had *so* [emphasis added] much time on our hands and now a lot has changed. I think there were more people involved in the garden then. People had time. People are in school now, they work”.

Participants F, G, H and D reflected on the limits to the time available for active participation in gardening. Participant D shared that another resident who had been granted international protection and left DP told her that “when she moves out, she can give me a space [raised bed] where she grows. But I didn’t have the time…sometimes in the evening, during the summer, she and her daughter would go into the garden and work. It was fine for her…But I didn’t have the opportunity to grow my own things in the garden…it was just a time thing”.

### 3.2. Space to Breathe, Space to Be

Participants C, D, F, G and H surfaced alternate ways that access to green space outside of international protection accommodation can enhance well-being and support recovery from trauma. Participant C said “I like coming out from my room sometimes in the garden to refresh my mind…you can see out and then you can just refresh your memories…we are all closed in our room, the space is not enough for us here. So, when you come out… [into] the garden, you still feel you have good memories, and you do not over stress yourself any more”. Participants D, E, F, G and H regard the garden as an autonomous space away from the scrutiny and overcrowding inside DP. Participant E clarified that the garden is “separate…it’s easy to come in, you don’t feel you are intruding in people’s space. It’s a neutral place outside…that it is actually separated from the lodge, that helps, its far enough”.

Open space in the garden can also help mitigate conflicts that may emerge in the congregated space inside DP. Participant C feels that she can express herself more authentically outside. She said “sometimes you want to make calls in our rooms…but there is no room…I am very loud in talking and in the next room [another resident] is making calls. So, when you come to the garden, you can shout, you can talk the way you feel like”. She also reflected that sharing time outside together can encourage socialisation among residents and promote greater harmony. She shared that “we have our friends, we don’t want them to come into our room. We can bring them to the garden and have something to eat and spend time together…I think it is a very lovely place”.

Participants C, D, F, G and H all use the garden dome regularly and find it has enhanced DP residents’ ability to enjoy outside space for more months of the year. Participant E recalled, “when I was still here the dome wasn’t built so you couldn’t have as much time [outside]. It’s become a place to meet, it’s a social place…since I’ve been out [of living in DP], I’ve been in here more than when I was in…I am always invited…even if you move out, you’re still part of this community…it caters for everybody. The kids are able to play in [the dome], adults are able to come and socialise in here. They have incorporated every age group”.

Participant I, a woman who had recently been allocated a room for her and her baby daughter in the Clonakilty DP centre, captured how access to green space supports well-being. She said “there are times…you are in a whole of emotions of your own when you are in that room. So, you just want to go out, breathe the fresh air, see people, talk to people. Even just staring at trees sometimes just give you a different vision of things. And for a moment you just forget you are coming from your room that is all soaked up with everything you have in our mind. I think the garden is doing a very great job, for me”. Participant I spoke about benefitting from activities going on in the garden during the summer and remarked that “when you come here…you will be interested because things are different from what you used to do. You’ll laugh differently, you can feel this lift within you…that you should do this more often”.

Participant J reflected on living at the DP centre in one small, shared family room accommodating her, her husband, her young daughter and her son who has special needs. Living in DP for several years, her family experienced a range of care deficits, which have taken a toll on them. She demonstrated a journey towards resilience, recalling “it’s difficult, you’re not mentally stable all the time in here. For me in here…most of the days it was very bad before. But now I manage everything…I become more strong than before”. She credits the Friends’ support workers and the activities they offer as a key factor in her ability to cope better. Participant J said “they are good. They are always open to come and talk with everyone…They are helping me, they are giving lots of therapy…like art therapy or sometimes we go to the forest, walking, it’s like nature healing therapy.” Participant J spends a lot of time in the garden with her children which supports her sense of well-being. She shared “I enjoy the nature. Like if I sit down and look at the trees…I feel something. It’s nice, I take the nature, the beautiful. I want to take this inside me [gestures her hands towards her heart]. Like peace and quiet”.

### 3.3. Multigenerational Benefits of Green Space

Participant J also revealed how crucial the space outside their crowded room is for both her and her children. She spoke about the benefits that she believes her children gain from being outdoors. She said “when my daughter and my son come here and play I feel like they need it. To touch the nature, touch the soil…It develops their brain [as] I know how I developed myself [growing up in the Global South close to nature]…I bring them mostly every day…Or a playground or garden, this kind of place like open place…I know they really need it”.

Participants in the focus group discission spoke about forms of care their children access within the garden. Participant F echoed that, though she is not actively engaged in gardening, she appreciates the benefits her child gains in the garden. She said “I’m not really involved in the garden, but my daughter really loves it. She is always here. She really loves gardening…They are always watering. I think it is good for them”.

Participants C, D, F, G and H commented that children growing up in DP benefit from access to autonomous space outside the rooms they share with their family. Participant D said “it’s helping them mentally because they are kids, they won’t bring other kids in the room because it is just one room…And sometimes they go to the garden, the garden is a lovely place…They are always happy, they look forward to go out. Its ok, outside of the box of the lodge”. Moreover, Participants C, D, F, G and H expressed that their own sense of well-being is related to how their children are feeling. They feel more relaxed when their children are having care needs met by the Friends’ support workers or are enjoying the activities offered in the garden. In this way, care delivered for and with children also honours their mothers’ need for space to rest and have time alone to cope with post-migration stressors.

## 4. Discussion

This research identifies forms of giving and receiving care through human connections with nature. In our anthropocene era, where climate crisis is a recognized driver of forced migration, there is an urgent need to identify solidaristic contributions to ‘universal care’ and take joint responsibility for ‘engaging with and caring about the flourishing of other people and the planet’ [17]. Themes emerging from our explorations with asylum seekers who spend time in the Clonakilty Community Garden reveal a *landscape of care,* resonatingwith the)health geography concept of ‘complex embodied and organizational spatialities that emerge from and through the relationships of care’ [38]. Furthermore, as the findings demonstrate interactions between people, culture and place they contribute to an examination of what good care means culturally and the idea of *cultural landscapes of care* [39].

The various forms of care identified in this garden help underscore how such a setting provides multiple opportunities for adults and children to benefit from individualised care tailored to their needs for psycho-social support and the changing contexts of the DP system. This contributes to an understanding of people living in the institutional setting of DP not as units or a dehumanised collective, but rather as individuals living with the cumulative effects of pre- and post-migration stress which can be alleviated somewhat with careful interventions. Participants demonstrate how access to green space provides a safe place to process trauma, allowing vulnerable forced migrants to enhance their sense of subjective well-being through connecting with nature and just having space to breathe.

Building on earlier observations of the ‘food activism’ [30] occurring when the raised beds in the garden were productive, our findings contribute to an understanding of the changing nature of life within Ireland’s international protection process. The emergence of asylum seekers’ right to employment and enhanced community integration resulted in less time for a gardening intervention that helped fill time. Nevertheless, the green space remains crucial as an autonomous place away from the institutionalised confines of DP, where asylum seekers can assert their right to privacy.

The multiple benefits from the support offered by a small community-based organisation honours the need for parents living in DP to have time alone to cope with pre- and post-migration stress and provides safe space for vulnerable migrant children to play outside [7,8,16]. Moreover, the open and collective space of a garden extends forms of solidarity and connection with the local community which can help overcome the isolation asylum seekers commonly face. This suggests a model of better care delivered outside of Ireland’s international protection accommodation system.

We observed that the ongoing engagement of children in gardening and nature-based activities allows parents to have time for rest in the knowledge that their children are being cared for and able to engage with nature in a safe and protected environment. By actively gardening, the children have opportunities to learn basic growing skills and experience the joy of harvesting their own produce and flowers, which resonates with evidence that exposure to nature nurtures children’s feelings of accomplishment and can develop their confidence [23]. As asserted in a recent review of the potential of gardens to be integrated into school-based Social and Emotional Learning initiatives, gardening can support children’s emotional needs in a safe environment [40]. As an addition to the established community garden, the sensory garden is an affordance offering a therapeutic experience for vulnerable migrants of all ages who have experienced trauma [41,42].

Participant accounts and observation suggest that autonomy and ownership of the garden are essential. Our observations imply that when vulnerable migrants can see a place as ‘their’ space it can confer lasting connections to people and planet. Moreover, directly involving the children in the planning and development of the sensory garden seems to have encouraged a sense of stewardship of nature and pride in what they came to see as ‘their’ space. We find that some former residents of the DP centre who have moved into the local community return for events held in the garden and thereby retain connections to people with a shared lived experience as they continue to develop their social network and life in Ireland. Importantly, care for this garden requires ongoing support, solidarity and voluntary effort, with some reliance on a small community-based organisation to identify how the green space can best be utilised to meet the ongoing and changing care needs of the population they are mandated to support. Our findings suggest that enhancing access to green space is one potential solution which could have protective effects for people seeking international protection [16] in an era when time, space and tolerance for community integration may be contracting in the EU.

We recognise certain limitations of the findings, particularly as we convey participants’ subjective statements about improvements to well-being, ability to cope with institutional confinement and uncertainty, and mitigation of the impacts of trauma and stress which are not measured through a specific intervention. Ethical approval which allowed the inclusion of adults in our sample resulted in the inability to conduct research directly with children, which results in over-reliance on secondary observations from parents, a researcher and a support worker. By demonstrating connections with similar findings from other settings we attempt to validate our participants’ experiences and suggest future directions for research using proven horticultural therapy interventions to be undertaken among international protection applicants in Ireland. Furthermore, our findings resonate with evidence of the positive health benefits of other nature-based activities that young asylum seekers living in the Clonakilty DP Centre have participated in such as a ‘surf therapy’ programme intended to overcome barriers to accessing coastal ‘blue space’ and enhance social, emotional and physical resilience through surfing [43]. Future research focused on children’s experiences of green spaces would yield important information from their own perspectives.

The initial research plan envisioned that a member of the research team could actively garden alongside adult asylum seekers to develop rapport based on care ethics such as trust and solidarity and explore who would be interested in participating in a focus group or one-to-one interview. After the first visit to the garden, the Friends clarified that the allotments were not being utilised by adults currently resident in the DP centre anymore, as now they had generally accessed the labour market. Thus, we maintained flexibility in using methods appropriate to a dynamic context, but we may have secured additional participants with a different approach.

We acknowledge that our findings could be regarded as representing a context where vulnerable migrants can easily self-regulate, but we are mindful of the precarity within international protection regimes. We note that the failure of Ireland’s vision to end the institutional model will soon be eclipsed by legislation, influenced by anti-migration discourse, that will change the future experiences of forced migrants throughout the EU.

## 5. Conclusions

This research contributes to the understanding of the potential of enhancing universal access to safe and inclusive green space and forms of horticultural therapy to support the health and well-being of vulnerable forced migrants. It brings attention to the landscapes of care which can thrive outside institutional structures which can help mitigate some of the damage which drives forced migration and is compounded in international protection settings.

This research adds to the evidence of the benefits of engaging vulnerable migrants in nature-based activities and demonstrates that the well-being of asylum seekers can be enhanced by simply having space outside of institutional living to decompress and relax by connecting with nature. Mindful of the extreme limits within and beyond international protection accommodation experienced during COVID lockdowns, the findings suggest that facilitating access to green space is an underexplored mechanism that could enhance the care of people often confined in institutional structures.

## Figures and Tables

**Table 1:** Participant information.

Participant	Gender	Living in DP *	Research Method
A	Male	Yes	Interview (online)
B	Female	Yes	Interview (online)
C	Female	Yes	Focus Group
D	Female	Yes	Focus Group
E	Female	No	Focus Group
F	Female	Yes	Focus Group
G	Female	Yes	Focus Group
H	Female	Yes	Focus Group
I	Female	Yes	Interview (in person)
J	Female	Yes	Interview (in person)

* at time of research.

**Table 2:** Research questions generating themes emerging from data analysis.

Research Method:	Research Question:
Interviews (online)	How do asylum seekers give meaning to care in their everyday lives, both as receivers and givers of care?
Focus group and in-person interviews	What are your thoughts on how care connects to what takes place in this community garden?

## Data Availability

The raw data supporting the conclusions of this article will be made available by the authors on request.

## Abbreviations

The following abbreviations are used in this manuscript:

DP	Direct Provision and Dispersal
EU	European Union
COVID-19	Coronavirus Disease 2019
COREQ	Consolidated criteria for reporting qualitative research
